# A model of electrophysiological heterogeneity in periglomerular cells

**DOI:** 10.3389/fncom.2013.00049

**Published:** 2013-04-26

**Authors:** Praveen Sethupathy, Daniel B. Rubin, Guoshi Li, Thomas A. Cleland

**Affiliations:** ^1^Computational Physiology Laboratory, Department of Psychology, Cornell UniversityIthaca, NY, USA; ^2^Department of Psychology, Cornell UniversityIthaca, NY, USA

**Keywords:** olfactory bulb, computational model, acetylcholine, juxtaglomerular neurons, NEURON simulator, glomerulus

## Abstract

Olfactory bulb (OB) periglomerular (PG) cells are heterogeneous with respect to several features, including morphology, connectivity, patterns of protein expression, and electrophysiological properties. However, these features rarely correlate with one another, suggesting that the differentiating properties of PG cells may arise from multiple independent adaptive variables rather than representing discrete cell classes. We use computational modeling to assess this hypothesis with respect to electrophysiological properties. Specifically, we show that the heterogeneous electrophysiological properties demonstrated in PG cell recordings can be explained solely by differences in the relative expression levels of ion channel species in the cell, without recourse to modifying channel kinetic properties themselves. This PG cell model can therefore be used as the basis for diverse cellular and network-level analyses of OB computations. Moreover, this simple basis for heterogeneity contributes to an emerging hypothesis that glomerular-layer interneurons may be better described as a single population expressing distributions of partially independent, potentially plastic properties, rather than as a set of discrete cell classes.

## Introduction

Olfactory bulb (OB) periglomerular (PG) cells are heterogeneous with respect to several features, including morphology, synaptic connectivity, patterns of protein expression, transmitter identity, transcription factor expression, and electrophysiological properties (Kosaka and Kosaka, 2005, 2012; Allen et al., 2007; Parrish-Aungst et al., 2007; Kiyokage et al., 2010). Interestingly, while subclasses of PG cells can be defined with respect to each of these features, the subclasses based on different features generally do not correlate—for example, knowing about a given marker expressed in a PG cell generally does not enable conclusions about its morphological or synaptic properties, its neurotransmitter, or its expression profile with respect to other molecular markers. Occasionally, however, correlations between specific features are observed. For example, while calbindin is expressed in a variety of OB juxtaglomerular interneurons including PG cells and superficial short-axon (sSA) cells (Kosaka and Kosaka, 2010), calbindin-positive PG cells in rats have been reported to receive no direct input from the olfactory nerve (Toida et al., 1998), indicating that calbindin is not expressed in the morphologically defined olfactory nerve-driven subtype of PG cell (PGo; Shao et al., 2009). As another example, only morphologically bipolar PG cells appear to express nicotinic cholinergic receptors in mice (Castillo et al., 1999). This pattern of mixed feature correlations suggests that PG cell heterogeneity may arise from multiple distinct and interacting processes rather than representing a discrete hierarchy of defined cell classes.

The electrophysiological properties of PG cells also are heterogeneous and defy easy categorization into discrete classes (Puopolo and Belluzzi, 1998; McQuiston and Katz, 2001; Shao et al., 2009). For example, PG cells have been grouped into subtypes based upon different patterns of potassium conductance expression (Puopolo and Belluzzi, 1998) as well as on whether or not they exhibit bursting properties based on a low-threshold calcium spike, as opposed to one of a range of non-bursting responses to somatic inputs (McQuiston and Katz, 2001). However, these physiological subtypes do not correlate with neuronal morphology, again reflecting the pattern of mixed feature correlations underlying PG cell heterogeneity. A further possibility is that the properties of any given cell may not be fixed, but plastic, enabling interneuronal response properties to be regulated in response to changing conditions or neuromodulatory state. Toward this end, we sought to build a biophysically-based reduced compartmental model of the PG cell in which the diverse electrophysiological response properties of PG cells that have been observed experimentally by McQuiston and Katz (2001) each could be evoked by varying the expression levels (relative conductances) of different ion channels, without modification of their kinetic properties or relative spatial distributions within the neuron. Specifically, McQuiston and Katz (2001) recorded from 21 presumptive PG neurons (in addition to recordings from other juxtaglomerular cells) and found them to exhibit diverse response profiles, from simple non-accommodating or accommodating spike trains to calcium T current-dependent low-threshold spikes (LTSs) crowned by single or bursts of sodium action potentials, along with some rarer responses. We here present a core PG cell model capable of replicating the diverse responses evoked under current clamp by McQuiston and Katz (2001) and suitable for extension pursuant to more specific simulation goals. Specifically, we replicate the various PG cell response profiles presented by these authors within a single computational model, modestly varying the conductance ratios of membrane channel classes in order to produce the diversity of responses exhibited by PG cells.

## Materials and methods

### Model architecture

**Figure 1:**
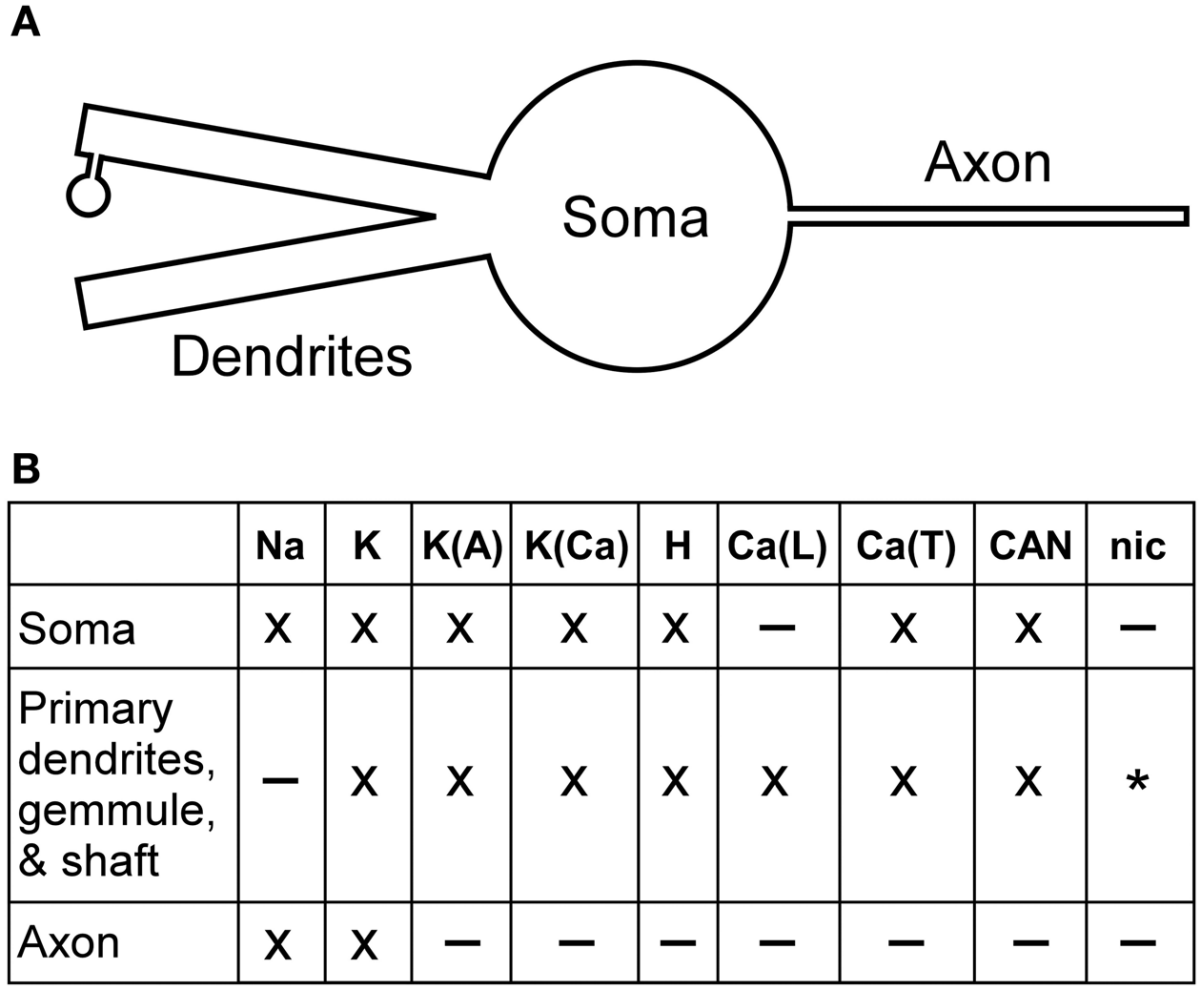
**Periglomerular cell model. (A)** Schematic representation of six-section PG cell model, comprising one gemmule (receiving synaptic input), a gemmule shaft, two dendrites (one to which the gemmule is attached), a soma, and an axon. **(B)** Expression profile of different channel types within model sections. Channel types marked as present in a given section (×) may have zero conductance under certain parameter sets (Table 3); channel types marked as absent in a given section (–) were always absent. The nicotinic cholinergic receptor channel (*G*_nic_), an ohmic cation channel, was expressed only in the gemmule compartment (see text for details).

The PG cell model was implemented in the NEURON simulation environment (Hines and Carnevale, 1997) and was shaped by four principal objectives (Rubin and Cleland, 2006). First, it should represent cellular properties with an accuracy and precision adequate for its intended purposes. Second, it must be reduced in complexity for computational efficiency, such that it can be usefully incorporated into moderately-sized network simulations. Third, it should be reasonably compartmentalized, so that elements of the model can be upgraded or redesigned as required without rendering the revised model incompatible with its predecessors. Fourth, unconstrained variables should be minimized, as they can improve apparent fits to data while reducing models' predictive value. Consequently, as this model is intended as a baseline model for simulations incorporating PG cell heterogeneity, six sections were implemented: a soma, two equivalent dendrites, a gemmule (spine) shaft and gemmule body attached to one of these dendrites, and an axon stub (Figure 1). The separate gemmule sections are relevant to olfactory nerve-driven PGo cell models in which feed-forward olfactory nerve input onto the PGo gemmule results in local neurotransmitter release onto the dendrites of other OB neurons, a property thought to underlie odor similarity-dependent computations in the OB (Cleland and Sethupathy, 2006; Cleland, 2010). The axon compartment, appropriately extended, is relevant to the modeling of PG cell interglomerular projections, and potentially to the construction of an sSA neuron model. The multiple dendrites are relevant to modeling studies of dendrodendritic interactions across PG cells. In the present modeling study, we emphasized replication of current-clamp responses measured from PG cell somata in an OB slice preparation (McQuiston and Katz, 2001).

### Model morphology and mechanisms

The geometric parameters of the cell were derived from Pinching and Powell (1971). Specifically, the soma was 8 um in length and diameter; all other model sections were 1 um in diameter. The gemmule and shaft compartments were 1 um in all dimensions. The axon stub was modeled as 50 um long, and each dendrite as 20 um long. Passive membrane capacitance was 1.2 uF/cm^2^. Additional passive parameters are listed in Table 1. The modeling of cable properties for appropriate compartmentalization was governed by NEURON's d_lambda rule; accordingly, the axon comprised three compartments whereas all other sections were modeled as single isopotential compartments. Increasing compartmentalization by factors of 3 or 9 produced identical results.

**Table 1:** **Passive membrane parameters**.

**Parameter**	**Value**
*C*_m_	1.2 μF/cm^2^
*R*_axial_	173 ohm-cm
*R*_in_(soma)	775 ± 55 MΩ
*E*_leak_	−70 mV^*^
*E*_Na_	+50 mV
*E*_K_	−85 mV
*E*_Ca_	Variable
*E*_H_, *E*_CAN_	0 mV
*E*_nic_	+3.2 mV

Input resistance R_in_ was measured at rest among each of the different current configurations and averaged. E_Ca_ varied dynamically with the internal perimembrane calcium concentration, from +110 to +120 mV at rest down to as low as +38 mV in the soma and +20 mV in the primary dendrite during low-threshold calcium spikes. The leak reversal potential E_leak_ was −70 mV in all cases except Figure 1A, for which it was −55 mV; see text for details.

We utilized hyperpolarization-activated cation current (*I*_H_) kinetics derived from PG cell recordings (Cadetti and Belluzzi, 2001). For other channel types, we adapted existing kinetic models as follows: a fast sodium spike current (*I*_Na_) and delayed rectifier potassium current (*I*_K_) (Destexhe et al., 1998), an A-type potassium current (Migliore et al., 1995), a low voltage-activated T-type calcium current [*I*_Ca(T)_] (Destexhe et al., 1996), an L-type high voltage-activated calcium current [*I*_Ca(L)_] (Carlin et al., 2000; Markaki et al., 2005), a calcium-dependent non-specific cation current (*I*_CAN_), a calcium-dependent potassium current [*I*_K(Ca)_] (Destexhe et al., 1994), and a current noise source (David et al., 2008). The noise source constituted white noise (bandwidth 0–4 kHz, standard deviation 50 fA) colored by convolution with a single exponential function (τ = 5 ms). Perimembrane calcium concentration after calcium influx (within 0.1 um of the membrane) was modeled as decaying exponentially to a basal level via diffusion into the cell volume, using a mechanism based on that of Destexhe and colleagues (1998). Simulation kinetics assumed a temperature of 23°C, consistent with the temperature at which the relevant slice recordings were performed (McQuiston and Katz, 2001).

Membrane current kinetics were implemented in NMODL (Hines and Carnevale, 2000) and were identical across all model sections for each of the channel types implemented in the model (Table 2). However, the levels of expression of each current mechanism (i.e., their maximum conductances) were varied to evoke different electrophysiological response properties in model simulations (Table 3). The current *I*_*x*_ through each channel was determined at every time step by the Hodgkin–Huxley formalism (Hodgkin and Huxley, 1952):

$$\begin{array}{l} \;I_{x}=\left(G_{\text{max}}\right)\left(m^{\text{a}}\right)\left(h^{\text{b}}\right)\left(V_{m}-E_{x}\right)\\ dm/dt=\Phi \left(m_{\infty }-m\right)\text{​}/\tau_{m}\\ dh/dt=\Phi \left(h_{\infty }-h\right)\text{​}/\tau_{h} \end{array}$$

where, for each channel type *x, G*_max_ defines the maximum conductance density of that channel in the designated compartment (Table 3), *m* and *h* are the activation and inactivation gating coefficients (of order *a* and *b*, respectively), *V*_*m*_ is the membrane potential within that compartment, *E*_*x*_ is the net reversal potential of the permeant ion(s), and Φ is the temperature coefficient (Table 2). Each gating coefficient was calculated from a differential function of its steady state (∞) and time constant (τ), both of which were functions of voltage. The gating coefficients of the *I*_CAN_ and *I*_K(Ca)_ currents also were functions of the internal perimembrane calcium concentration, which was governed by calcium influx and by the constitutive diffusion rate of calcium out of the 0.1 um deep perimembrane region.

**Table 2:** **Kinetic equations for PG cell model currents**.

**Current type**	**Gating variable**	**ϕ**_***x***_	**α**_**x**_, ***x***_**∞**_	**β**_**x**_, **τ**_**x**_ **(ms)**	**Citation**
*I*_Na_	*a* = 3	0.24	$\alpha_{m}=\frac{0.32(V+39)}{1-\exp(-(V+39)/4)}$	$\beta_{m}=\frac{-0.28(V+12)}{1-\exp((V+12)/5)}$	Destexhe et al., 1998
			*m*_∞_ = α_*m*_/(α_*m*_ + β_*m*_)	τ_*m*_ = 1/(α_*m*_ + β_*m*_)	
	*b* = 1	0.24	α_*h*_ = 0.128exp(−(*V* + 35)/18)	$\beta_{h}=\frac{4}{1+\exp(-(V+12)/5)}$	
			*h*_∞_ = α_*h*_/(α_*h*_ + β_*h*_)	τ_*h*_ = 1/(α_*h*_ + β_*h*_)	
*I*_K_	*a* = 4	0.24	$\alpha_{m}=\frac{0.032(V+37)}{1-\exp(-(V+37)/5)}$	β_*m*_ = 0.5exp(−(*V* + 42)/40)	Destexhe et al., 1998
			*m*_∞_ = α_*m*_/(α_*m*_ + β_*m*_)	τ_*m*_ = 1/(α_*m*_ + β_*m*_)	
*I*_K(A)_	*a* = 1	0.46	α_*m*_ = exp(−0.118(*V* + 33.6))	β_*m*_ = exp(−0.071(*V* + 33.6))	Migliore et al., 1995
			*m*_∞_ = 1/(1 + α_*m*_)	τ_*m*_ = 50 β_*m*_/(1 + α_*m*_)	
	*b* = 1	0.46	α_*h*_ = exp(0.157(*V* + 83))	β_*h*_ = exp(0.157(*V* + 83))	
			*h*_∞_ = 1/(1 + α_*h*_)	τ_*h*_ = 12.5 β_*h*_/(1 + α_*h*_)	
*I*_K(Ca)_	*a* = 2	1.12	$m_{\infty }=\frac{{\left[Ca\right]}_{i}^{2}}{6.25e^{-4}+{\left[Ca\right]}_{i}^{2}}$	τ_*m*_ = max{0.021/(6.25*e*^−4^ + [*Ca*]^2^_*i*_), 0.1}	Destexhe et al., 1994
*I*_H_	*a* = 1	0.35	$m_{\infty }=\frac{1}{1+\exp((V+80)/10)}$	$\tau_{m}=\frac{1176.5\exp((V+65)/23.5)}{1+\exp((V+65)/11.8)}$	Cadetti and Belluzzi, 2001
*I*_Ca(L)_	*a* = 2	1	$m_{\infty }=\frac{1}{1+\exp(-(V+30)/6)}$	τ_*m*_ = 20	Carlin et al., 2000; Markaki et al., 2005
	*b* = 1	1	*h* = *h*_∞_ = 1.245/(1.245 + [*Ca*]_*i*_)		
*I*_Ca(T)_	*a* = 2	0.85	$m_{\infty }=\frac{1}{1+\exp(-(V+49)/7.4)}$	$\tau_{m}=3+\frac{1}{\exp((V+24)/10)+\exp(-(V+99)/15)}$	Destexhe et al., 1996
	*b* = 1	0.90	$h_{\infty }=\frac{1}{1+\exp((V+77)/5)}$	$\tau_{h}=85+\frac{1}{\exp((V+45)/4)+\exp(-(V+404)/50)}$	
*I*_CAN_	*a* = 2	1.12	$m_{\infty }=\frac{{\left[Ca\right]}_{i}^{2}}{1e^{-4}+{\left[Ca\right]}_{i}^{2}}$	τ_*m*_ = max{1/(2*e*^−3^ + 20[*Ca*]^2^_*i*_), 0.1}	Destexhe et al., 1994

Mechanisms were adapted from previously published models as cited.

**Table 3:** **Active channel conductance densities (S/cm^2^)**.

**Figure**	**PG response type**	***G***_**Na**_	***G***_**K**_	***G***_**K(A)**_	***G***_**K(Ca)**_	***G***_**H**_	***G***_**Ca(L)**_	***G***_**Ca(T)**_ **(soma)**	***G***_**CAN**_
2A	Non-accommodating simple spike train	0.02	0.01	0.01	−	0.002	−	−	−
2B	Accommodating simple spike train	0.01	0.001	0.005	−	0.001	−	4.00e-4	−
2C	Single spike	0.01	0.002	0.02	−	−	−	2.00e-4	−
2D	Irregular spiking (rare)	0.02	0.01	0.01	−	0.005	−	1.00e-4	−
3A	LTS with single AP	0.01	0.1	0.1	−	3.58e-5	−	0.005	−
3B	LTS with AP burst	0.011	0.075	0.025	−	3.58e-5	−	0.002	−
3C	AP burst with extended plateau potential	0.004	0.007	0.001	0.001	0.0005	0.001	0.0001	0.00128
3D	AP burst with extended plateau potential	0.004	0.007	0.001	0.001	0.0005	0.001	−	0.00128
3E	AP burst with extended plateau potential	0.004	0.006	0.001	0.001	0.0005	0.001	0.0001	0.00128

**Figure 2:**
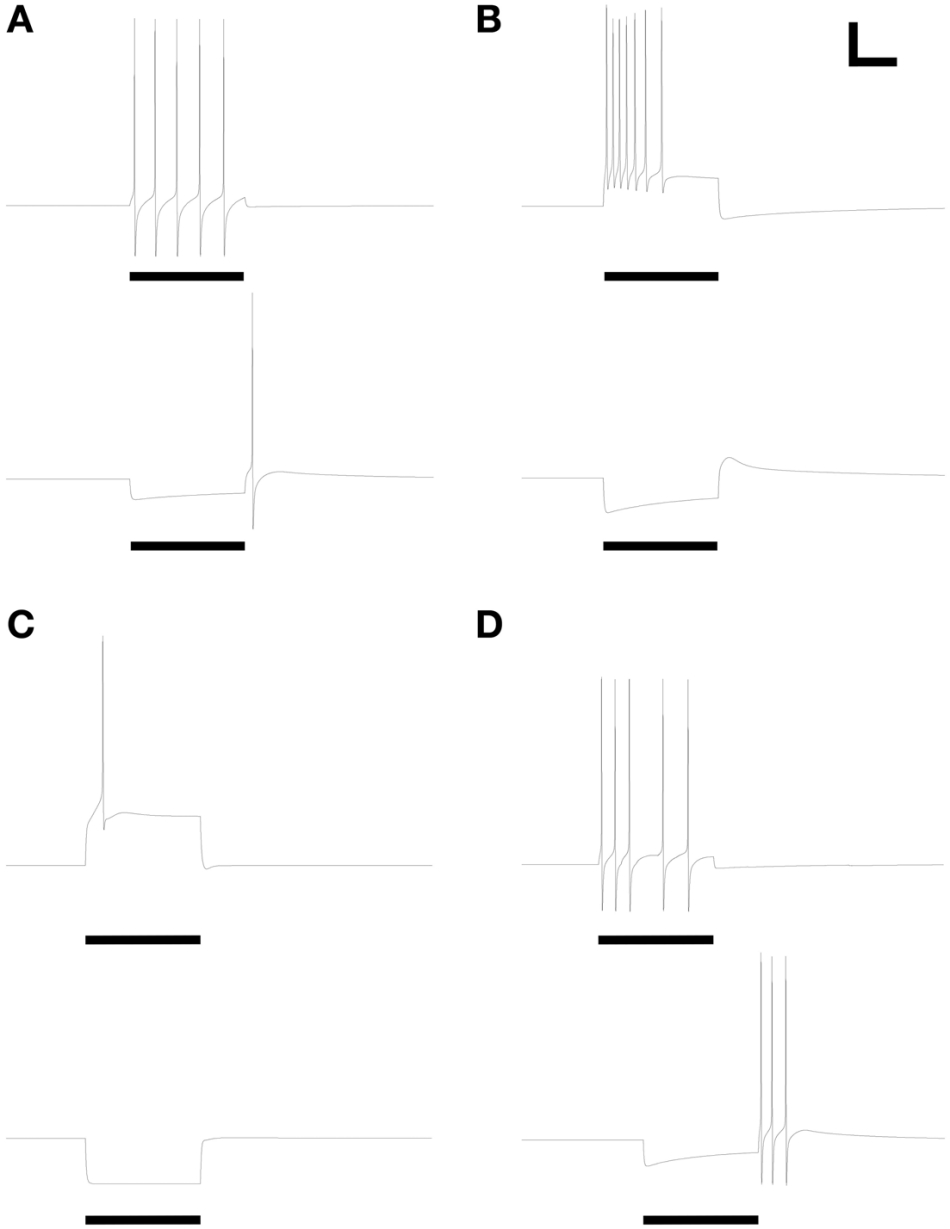
**Model periglomerular cells display a range of simple spiking properties, each closely resembling their counterparts in Figure 1 of McQuiston and Katz (2001).** Maximal conductance values for each membrane mechanism differ between panels (Table 3) but are identical within each depolarized/hyperpolarized pair of traces. **(A)** Non-accommodating spike train response to 3.5 pA depolarizing current (upper panel) and the corresponding response of the same neuron to release from a 1.2 pA hyperpolarizing current (lower panel). **(B)** Accommodating spike train response to 22 pA depolarizing current (upper panel) and the corresponding response of the same neuron to release from a 22 pA hyperpolarizing current (lower panel). (C) Single spike response to 25 pA depolarizing current (upper panel) and the corresponding response of the same neuron to release from a 25 pA hyperpolarizing current (lower panel). **(D)** Irregular spiking response to 7.5 pA depolarizing current (upper panel) and the corresponding anode break burst response of the same neuron to release from a 20 pA hyperpolarizing current (lower panel). Stimulus durations were all 600 ms (horizontal bars). Scale bars for all panels: 20 mV, 250 ms.

Channel conductance densities were uniform across all model sections in which they were present at all (Figure 1B), except for the T-type low threshold calcium conductance, which was always expressed 5.667 times more densely in the dendritic and spine compartments than in the soma. Irregular spiking responses (Figure 2D) also required the addition of a current noise mechanism (see Materials and Methods).

### Computational methods and parameterization

All simulations were run in NEURON version 7.2 (http://www.neuron.yale.edu) (Hines and Carnevale, 1997, 2001; Carnevale and Hines, 2006). Timesteps were dynamically determined by NEURON's CVODE algorithm. The change in voltage in each compartment was calculated as the sum of all ionic currents, injected currents, and currents flowing from the neighboring compartments:

$$\begin{array}{l} C_{\text{m}}\left(dV_{a}/dt\right)=-I_{\text{leak}}-I_{\text{Na}}-I_{\text{K}}-I_{\text{K}(\text{A})}-I_{\text{K}(\text{Ca})}-I_{\text{H}}-I_{\text{Ca}(\text{L})}\\ \;-I_{\text{Ca}(\text{T})}-I_{\text{CAN}}-I_{\text{nic}}-\sum \left(I_{\text{adj}}\right)+I_{\text{inj}} \end{array}$$

where *C*_*m*_ was the membrane capacitance and *I*_*x*_ denotes particular channel-specific currents except as follows: *I*_leak_ represents the leak current, *I*_nic_ an ohmic cation current associated with the nicotinic cholinergic receptor channel, *I*_adj_ denotes currents from adjacent connected model sections, and *I*_inj_ denotes experimental current injection into the soma compartment. In the simulations presented here, membrane potential recordings were made from the soma in order to replicate experimental methods (McQuiston and Katz, 2001). Final parameterization was performed as described in Rubin and Cleland (2006). Parameters were additionally constrained so that the same parameter set produced both the depolarization-induced and hyperpolarization-induced responses recorded experimentally from the same neuron, consistent with the corresponding experimental figures (McQuiston and Katz, 2001).

## Results

### Response properties comprising simple spike trains

The expression levels of different membrane currents first were adjusted to replicate the experimentally observed range of simple (non-bursting) electrophysiological properties depicted in Figures 1A–D of McQuiston and Katz (2001). (The fifth simple response profile described in that work (Figure 1E in McQuiston and Katz, 2001) was never observed in PG cells (Table 2 in McQuiston and Katz, 2001) and hence was not modeled). Under the first parameter set (Table 3), depolarizing current evoked a non-accommodating series of action potentials (Figure 2A), whereas hyperpolarizing current evoked a modest H-current dependent sag and generated an action potential upon release (*anode break response*; Figure 2A). Higher levels of depolarizing activation evoked higher rates of non-accommodating action potentials, whereas stronger inhibition led to anode break responses evoking multiple action potentials. Other ratios of the same membrane conductances replicated the remaining simple responses of different PG neurons; specifically, trains of accommodating spikes upon depolarization with a prominent post-inhibitory rebound after hyperpolarization (Figure 2B), single spikes upon depolarization without significant sag or rebound after hyperpolarization (Figure 2C), or irregular spiking patterns upon depolarization with a prominent sag and rebound-evoked spike burst after hyperpolarization (Figure 2D).

All responses were generated by altering the relative expression levels of five membrane mechanisms (Table 3), with two exceptions. First, evoking a non-accommodating train of spikes (Figure 2A) required a relatively depolarized leak reversal potential (−55 mV, rather than the −70 mV used for all other figures; Table 1). Alternatively, activation of a nicotinic receptor-induced current—modeled as a 5 mS/cm^2^ ohmic cation conductance expressed in the single dendritic spine—changed accommodating and single-spiking neurons to instead produce non-accommodating spike train responses. In a network context, nicotinic neuromodulation of PG cells enhances their feed-forward inhibition of mitral cells, contributing to the sharpening of odor representations within OB (Li and Cleland, 2013). Restriction of nicotinic receptor channels to the spine compartment reflected the distribution of cholinergic synaptic inputs to PG neurons observed via choline acetyltransferase immunohistochemistry (Kasa et al., 1995), though similar results were obtained using a uniform expression of nicotinic receptor channels. Second, the irregular spiking patterns of Figure 2D, replicating a relatively unusual form of activity among recorded neurons (McQuiston and Katz, 2001), were produced with aid of a current noise model (David et al., 2008; see Materials and Methods). Such activity may reflect irregular synaptic inputs from unusually active neurons, possibly owing to damage during slice preparation.

### Response properties including calcium T current-dependent low-threshold spikes

**Figure 3:**
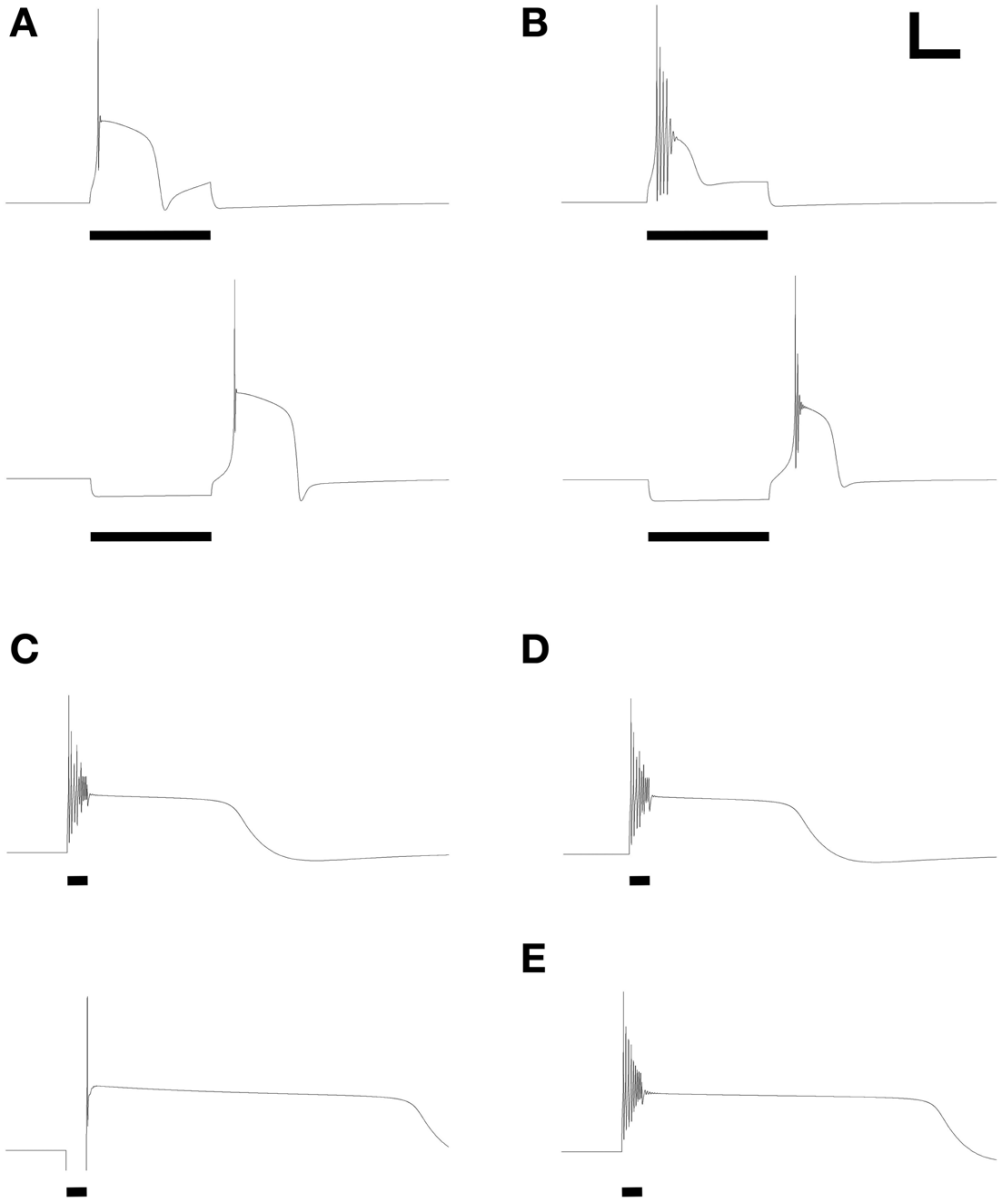
**Model periglomerular cells display low threshold calcium spikes or persistent plateau potentials.** Panels each closely resemble their counterparts in Figure 2 of McQuiston and Katz (2001). Maximal conductance values for each membrane mechanism differ between panels (Table 3) but are identical within each depolarized/hyperpolarized pair of traces. **(A)** Depolarizing current injection (10 pA, 600 ms) activated a low-threshold spike (LTS) with a single action potential (upper panel); release from 10 pA of hyperpolarizing current also evoked a similar LTS. **(B)** With modestly modified conductance ratios, injection of 10 pA depolarizing current (upper panel) or release from 10 pA of hyperpolarizing current (lower panel) evoked an LTS crowned with a train of decrementing spikes. **(C)** To replicate the rare persistent plateau potential response illustrated by McQuiston and Katz, additional current mechanisms were implemented. As in the original experimental results, depolarization (30 pA, 200 ms) led to a train of decrementing spikes followed by a plateau potential, whereas release from hyperpolarization (20 pA) generated only a single spike but also evoked a persistent plateau potential. **(D)** Elimination of the T-type calcium conductance had no appreciable effect on the depolarization-induced plateau potential. **(E)** Modest reduction of the delayed rectifier potassium current (Table 3) extended the duration of the plateau. Adjustment of *I*_CAN_ and *I*_K(Ca)_ conductances also could regulate plateau duration. Scale bars for panels **(A,B)**: 20 mV, 250 ms; for panels **(C–E)**: 20 mV, 500 ms.

Additional distinct combinations of membrane conductance densities (Table 3) evoked a family of responses based on calcium *T* current-dependent LTSs and directly corresponding to those experimentally evoked from PG cells in Figures 2A–C of McQuiston and Katz (2001) and also observed by Zhou and colleagues (2006). Depolarizing current injected into one such model neuron produced an LTS crowned with a single fast sodium spike, whereas injection of hyperpolarizing current produced an anode break response upon release leading to an LTS with similar properties (Figure 3A). A different ratio of currents generated an LTS response upon depolarization that produced a burst of decrementing spikes, and a similar LTS response upon release from hyperpolarization (Figure 3B). Both LTS responses resulted in substantially higher transient intracellular calcium concentrations in the model compared with non-LTS responses. Experimentally, these LTS-based responses were observed in PG cells twice as frequently as were the simple non-LTS spike trains (McQuiston and Katz, 2001). The repeated bursting observed in some JG neurons (Figure 5E of McQuiston and Katz, 2001) is a property of external tufted cells (Hayar et al., 2004)—a discrete, glutamatergic class of JG neuron—and was not incorporated in the present PG cell model. The effects of diverse JG membrane currents on LTS properties have been analyzed in detail by Masurkar and Chen (2011a,b).

### Response properties incorporating persistent plateau potentials

Finally, the model was able to generate a persistent plateau potential upon both depolarization and release from hyperpolarization (Figure 3C). This response was very rarely observed experimentally and therefore is poorly defined physiologically (McQuiston and Katz, 2001). In the model, this response profile required the inclusion of additional conductances beyond those that were capable of generating simple spiking responses and calcium LTSs (see next section; Table 3). The persistent plateau was not LTS-dependent; reducing the T-type calcium current to zero did not affect plateau duration (Figure 3D), whereas modestly reducing the potassium delayed rectifier current (Table 3) substantially extended the plateau (Figure 3E).

### Conductance timecourses

**Figure 4:**
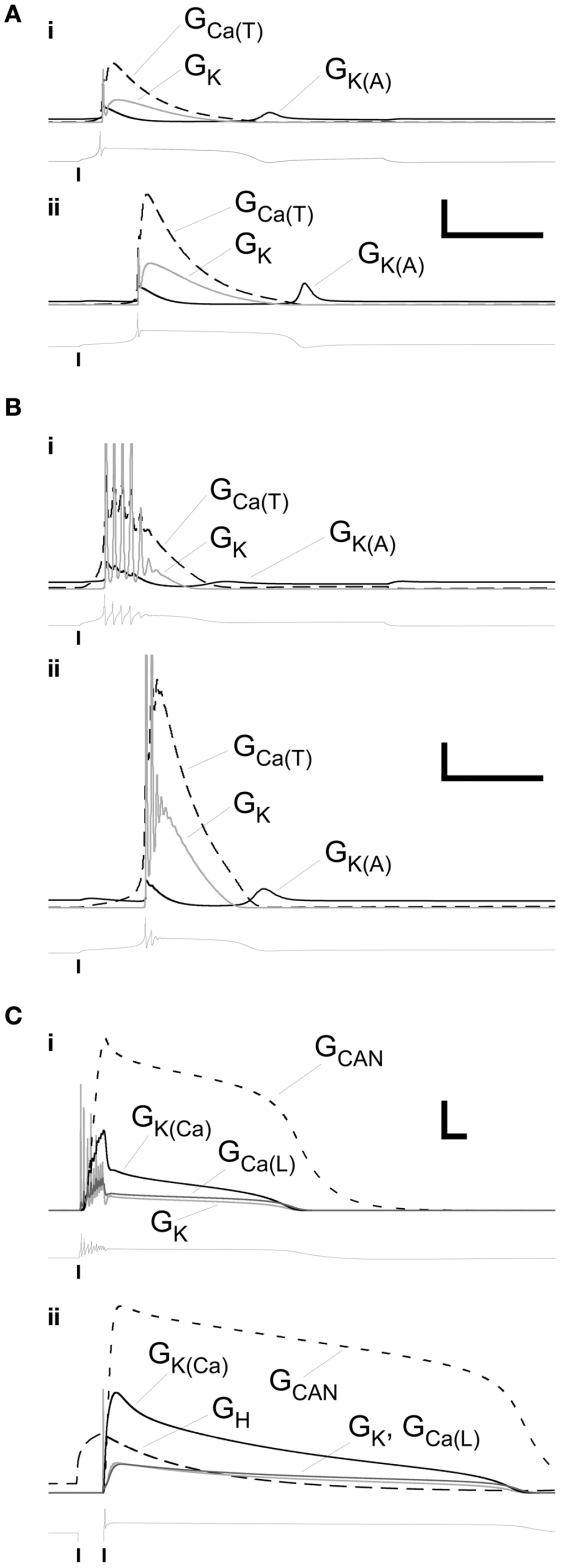
**Profiles of selected membrane conductances within primary dendrite during the LTS and plateau responses illustrated in Figures 2A–C. (A)** Conductance timeseries associated with the responses illustrated in the upper panel (depolarizing stimulus, (i) and lower panel (release from hyperpolarization), (ii) of Figure 2A. Scale bars: 2.0 mS/cm^2^, 200 ms. **(B)** Conductance timeseries associated with the responses illustrated in the upper panel (i) and lower panel (ii) of Figure 2B. Scale bars: 0.2 mS/cm^2^, 200 ms. **(C)** Conductance timeseries associated with the responses illustrated in the upper panel (i) and lower panel (ii) of Figure 2C. Scale bars: 0.2 mS/cm^2^, 200 ms. Copies of the voltage timeseries are displayed below each group of conductance profiles. Vertical lines denote the onset or offset times of injected currents.

We mapped the timecourses of the most important membrane conductances in the model to examine their interplay. The single-spike LTS, whether evoked by direct depolarization (Figure 4Ai) or by an anode break response (Figure 4Aii), depended strongly on a low-threshold T-type calcium conductance. Its timecourse was primarily determined by that of the Ca_(T)_ current itself, with a short post-LTS hyperpolarization punctuated by a modest deinactivation of the A-type potassium current. A roughly similar dynamical pattern of currents underlaid the LTS responses that included a decrementing burst of spikes, although the absolute dynamical levels of these conductances were reduced by roughly a factor of ten (Figure 4B; note vertical scale). Specifically, whereas the maximal conductances of voltage-dependent membrane currents in PG neurons evoking this response ranged from 25 to 110% of those in PG neurons evoking single-spike LTSs (Table 3), the dynamical conductances were on the order of 10–20% of those in the latter group. The overall dynamical dependence on the interactions among these different membrane conductances was, however, similar for both forms of the LTS-based response.

In contrast to the simple and LTS-based responses described above, the persistent plateau potential responses replicated in Figures 3C–E required the inclusion of additional conductances. Lacking constraining pharmacology from their uncommon observation in source data, we modeled them using an established plateau potential combination of a voltage-dependent calcium current, a calcium-dependent cation current, and a calcium-dependent potassium current (Table 3). These currents are present in JG neurons, though they are not important determinants of the more common LTS timecourses (Masurkar and Chen, 2011a,b) and hence were omitted from simple and LTS simulations to reduce the dimensionality of the model (Table 3). Calcium entering the cell in response to direct depolarization (Figure 4Ci) or in response to H current-dependent depolarization following release from a hyperpolarizing pulse (Figure 4Cii) activated both the cation conductance *G*_CAN_ and the potassium conductance *G*_K(Ca)_. The large cation conductance generated and maintained the plateau, declining along with the gradual decline in perimembrane calcium concentration until it could no longer be sustained. The calcium-dependent potassium conductance was primarily responsible for the termination of the burst of spikes following depolarizing input, but otherwise also followed the declining perimembrane calcium concentration. Unlike the much more common LTS responses, the role of the calcium *T* current in these persistent plateau potentials was negligible. Due to the absence of constraining pharmacological data regarding this response profile, it was not investigated further in the model.

### Pharmacology of low-threshold spiking responses

**Figure 5:**
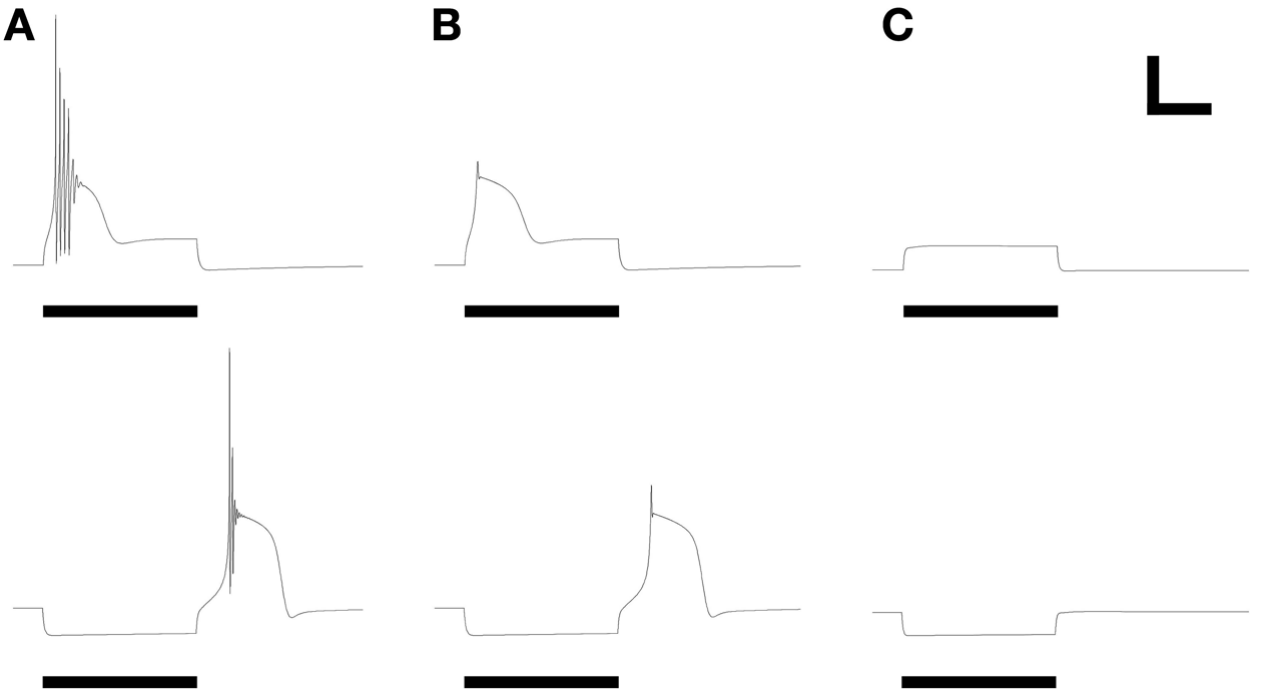
**Replication of LTS pharmacology.** Panels reflect their counterparts in Figures 3, 4 of McQuiston and Katz (2001). **(A)** LTS response under control conditions, using parameters of Figure 2B. **(B)** Evoked response after reducing the fast sodium conductance to zero, mimicking the application of intracellular QX314 or substitution of bath sodium with choline. **(C)** Evoked response after reducing the T-type calcium conductance to zero, mimicking the application of nickel ions or α-methyl-α-phenylsuccinimide (MPS). Stimulus durations were all 600 ms (horizontal bars). Scale bars: 20 mV, 250 ms.

The ionic basis of LTS responses in PG cells was experimentally determined using pharmacological manipulations. Specifically, blockade of sodium influx by choline replacement of external sodium or by intracellular infusion of the fast Na channel antagonist QX314 did not eliminate the LTS from PG cells (McQuiston and Katz, 2001). Similarly, in the model (Figure 5), reduction of the fast sodium conductance to zero prevented action potential generation but preserved the LTS response to both depolarizing current injection and release from hyperpolarization (Figure 5B). In contrast, reducing the T-type calcium current to zero (as if superfusing the tissue with nickel ions) completely blocked the LTS responses (Figure 5C), as demonstrated experimentally (McQuiston and Katz, 2001). The ionic basis for LTS responses in the model thus reflects those ascertained experimentally in PG neurons.

## Discussion

Network models incorporating specific biophysical elements of interest can produce useful predictions about the impact of these elements even if the implementation of other cellular properties, such as the cable properties of extensive neuritic arbors, is generic. Moreover, for purposes of understanding network properties, computational efficiency is critical, and it has been clearly established that morphologically reduced (oligocompartmental) models are capable of replicating the global response patterns of complex neurons, even when single-compartment models fall short (Davison et al., 2000; Rubin and Cleland, 2006; Li and Cleland, 2013). Within these constraints, the present model replicates the diverse physiological and pharmacological responses of PG neurons as observed experimentally. Pursuant to particular applications, the model architecture can be morphologically elaborated or further reduced with manageable effects on whole-cell performance.

### Interneuronal diversity in olfactory bulb: distributions or discrete classes?

We demonstrate computationally that each of the diverse simple and LTS-based responses observed experimentally in PG cells by McQuiston and Katz (2001) can be generated by relatively modest changes in the relative levels of expression of five membrane conductances without altering their kinetic properties or relative spatial distributions across the cell (Figures 2A–C, Figures 3A,B; Table 3; this summary excludes the noise model of Figure 2D and the rare persistent plateau potential responses of Figures 3C–E.). While the prevalence of each of these “common” physiological response profiles *in vivo* is not known, this simple basis for heterogeneity contributes to the emerging hypothesis that glomerular-layer GABAergic and dopaminergic interneurons may be better described as a single population expressing distributions of partially independent properties, rather than as a series of discrete cell classes (Kosaka et al., 1998).

PG cells have been subdivided into categories on the basis of morphology (bipolar vs. monopolar, axonless or with axon), extending dendrites to either contact the axons of primary olfactory sensory neurons (PGo, or “type 1”) or not (PGe, or “type 2”), marker expression (e.g., calbindin, calretinin, parvalbumin, nitric oxide synthase, nicotinic cholinergic receptor), and presumptive neurotransmitter (GABA, dopamine, both) (Kosaka and Kosaka, 2005, 2007, 2011; Shao et al., 2009). While there are occasional, possibly important correlations between certain feature sets (e.g., see Kosaka et al., 2001), generally these properties do not predict one another, appearing to be partially independent and forming a complex pattern of mixed feature correlations. Indeed, even sSA neurons, while exhibiting broad axonal projection patterns quite dissimilar from those of classically described PG cells (Aungst et al., 2003; Cleland, 2010), may not constitute a genuinely separate class of neurons. Like PG cells, sSA neurons are thought to utilize GABA and/or dopamine as neurotransmitters [though they were initially identified as glutamatergic; (Aungst et al., 2003; Brill et al., 2009)]. Most sSA neurons project axons to relatively proximate glomeruli, not unlike that nominal subclass of PG neurons that projects axons to one or a few neighboring glomeruli. The sSA cells that project long-distance axons to distant glomeruli are relatively uncommon (Aungst et al., 2003)—albeit essential to the small-world network computation proposed to mediate global feedback normalization (Cleland et al., 2007; Cleland, 2010)—suggesting that these sSA cells may constitute one tail of a distribution of morphological properties exhibited by a single heterogeneous population of interneurons, with the axonless form of “PG” interneurons constituting an opposing tail. Indeed, very recent work (Kiyokage et al., 2010; Marbach and Albeanu, 2011; McGann, in press) has begun to employ a revised nomenclature relabeling some traditionally-construed PG cells as sSA cells. Specifically, GAD67-expressing juxtaglomerular cells—which often coexpress tyrosine hydroxylase and include traditionally recognized sSA cells as well as many traditionally-construed PG cells—are being labeled as sSA (or simply SA) neurons in this nomenclature whereas GAD65-expressing PG cells retain the PG label. Both groups are heterogeneous with respect to other features, though GAD65-expressing PG cells do not exhibit the most broadly projecting of the multiglomerular morphologies. Whatever its merits, this revised nomenclature highlights the arbitrariness of discrete classification schemes for PG/sSA neurons. Moreover, with continuing study, increasing numbers of OB interneurons have been observed that violate the defining features of any of these nominal classes. For example, some nominal sSA neurons feature extrabulbar axonal projections, and some DA/GABAergic juxtaglomerular neurons project to the contralateral OB (Kosaka and Kosaka, 2008, 2011), a profile generally associated with the glutamatergic external tufted cells (Schoenfeld et al., 1985).

If this single heterogeneous interneuron population hypothesis is correct, then the fundamental experimental question shifts from “what are each of these cell types for?” to “what factors cause or require the expression of particular markers or properties within neurons of this [single] population?” That is, the observed pattern of mixed feature correlations suggests that PG cell heterogeneity may arise from multiple distinct and interacting processes rather than representing a discrete hierarchy of defined cell classes, and that certain nominal class markers may signal relatively transient neuronal states rather than durable identities. For example, while the calcium binding protein markers expressed heterogeneously among PG neurons have not typically been associated with particular functions, recent work has implicated calbindin-D28k, particularly, in some forms of synaptic plasticity (Schmidt, 2012; Westerink et al., 2012). Consequently, the presence of this marker in a subset of PG cells might identify those PG cells that have been recently synaptically modified. More broadly, the properties of any given PG cell may not be fixed, but plastic and separately regulated, thereby enabling interneuronal response properties to adapt in response to changing conditions or neuromodulatory state. The concept of discrete, static classes of OB interneuron may no longer serve.

### Conflict of interest statement

The authors declare that the research was conducted in the absence of any commercial or financial relationships that could be construed as a potential conflict of interest.
